# Automatic Synthesis of Panoramic Radiographs from Dental Cone Beam Computed Tomography Data

**DOI:** 10.1371/journal.pone.0156976

**Published:** 2016-06-14

**Authors:** Ting Luo, Changrong Shi, Xing Zhao, Yunsong Zhao, Jinqiu Xu

**Affiliations:** 1 School of Mathematical Sciences, Capital Normal University, Beijing, China; 2 Beijing Higher Institution Engineering Research Center of Testing and Imaging, Beijing, China; Chongqing University, CHINA

## Abstract

In this paper, we propose an automatic method of synthesizing panoramic radiographs from dental cone beam computed tomography (CBCT) data for directly observing the whole dentition without the superimposition of other structures. This method consists of three major steps. First, the dental arch curve is generated from the maximum intensity projection (MIP) of 3D CBCT data. Then, based on this curve, the long axial curves of the upper and lower teeth are extracted to create a 3D panoramic curved surface describing the whole dentition. Finally, the panoramic radiograph is synthesized by developing this 3D surface. Both open-bite shaped and closed-bite shaped dental CBCT datasets were applied in this study, and the resulting images were analyzed to evaluate the effectiveness of this method. With the proposed method, a single-slice panoramic radiograph can clearly and completely show the whole dentition without the blur and superimposition of other dental structures. Moreover, thickened panoramic radiographs can also be synthesized with increased slice thickness to show more features, such as the mandibular nerve canal. One feature of the proposed method is that it is automatically performed without human intervention. Another feature of the proposed method is that it requires thinner panoramic radiographs to show the whole dentition than those produced by other existing methods, which contributes to the clarity of the anatomical structures, including the enamel, dentine and pulp. In addition, this method can rapidly process common dental CBCT data. The speed and image quality of this method make it an attractive option for observing the whole dentition in a clinical setting.

## Introduction

Panoramic radiography and cone beam computed tomography (CBCT) are two common types of imaging techniques in modern dentistry [1–6]. Panoramic radiography projects the maxillae, mandible, nasal cavities, whole dentition and temporomandibular joints onto a single plane. A panoramic radiograph provides doctors with an overview of a patient’s oral health and the state of their teeth associated structures; however, the geometric distortion, blur and structural superimposition present in these images could influence clinical judgment. CBCT has been shown to be an effective imaging technique for the analysis of dental structures and pathologies, and images reconstructed with CBCT are consistent with the actual structures of the patients [2]. However, CBCT images cannot directly show the whole dentition which is essential for routine clinical purposes. Extracting panoramic radiographs from dental CBCT data would be beneficial for viewing the anatomical structures of the whole dentition, making diagnoses and conducting further research [7–14].

Recently, the creation of panoramic radiographs from dental CBCT data has been actively investigated. Tohnak et al. created the medial axis of the dental arch using thinning methodologies and computed the discrete Radon transform along normal lines perpendicular to the created medial axis [15]. While this method can show the whole dentition in the resulting image, some manual operations are required. To avoid human intervention, Akhoondali et al. proposed a fully automatic method of creating the mandibular curve and extracting the panoramic radiograph [16]. However, this method can only automatically handle open-bite shaped data, not closed-bite shaped data. Thereafter, Bing et al. improved Tohnak’s method to automatically generate panoramic radiographs, but this method was only tested with a mandibular CBCT dataset [17]. While Sa-ing et al. were able to automatically process all types of datasets, the whole dentition was only shown in the ray-sum panoramic radiograph and was significantly blurry [18]. Each of these studies initially created a curve following the form of the human dental arch and then projected the dental CBCT data along normal lines perpendicular to that curve. In these studies, the dental arch curve is used on all horizontal slices, forming a cylinder. As this method generates panoramic radiographs by projecting several cylinders onto a central cylinder, we named it the cylinder method. Because the angles between the long axis of incisors and the vertical direction are inconsistent, the cylinder method needs to augment the length of the normal line in order to show the whole dentition, resulting in a blurred image.

In this paper, we propose an automatic method for synthesizing panoramic radiographs from dental CBCT data without human intervention. This method extracts the 3D panoramic curved surface to describe the whole dentition, and then creates the panoramic radiograph by developing the 3D surface. The single-slice panoramic radiograph produced by this method can completely show the whole dentition without any manual operations and can clearly display the anatomical structures. Moreover, this method is also able to generate thickened panoramic radiographs; thus, more features, such as the mandibular nerve canal, can be shown.

## Materials and Methods

**Fig 1:**
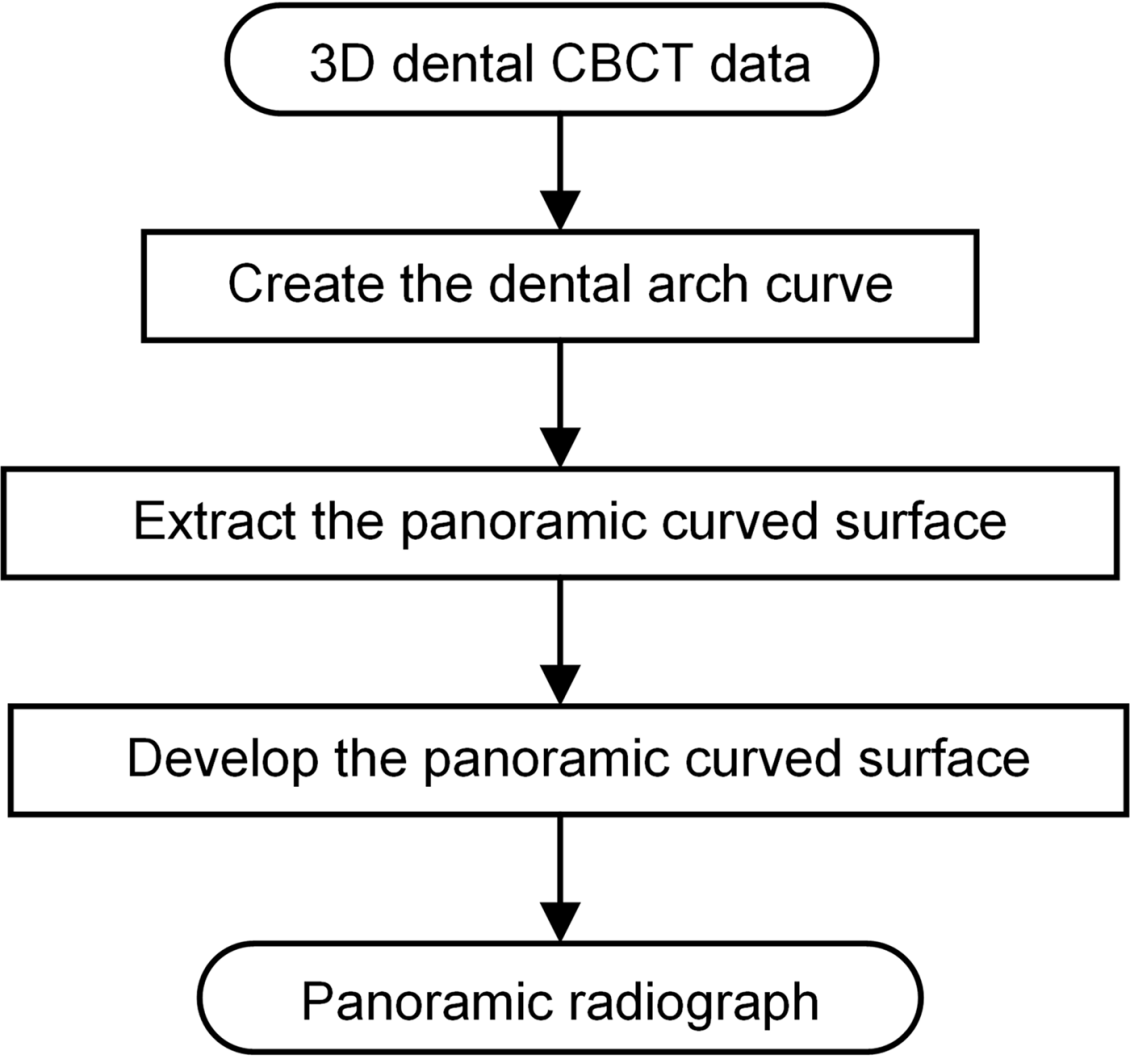
Block diagram of the proposed method.

To show the whole dentition in a single-slice panoramic radiograph, the extraction of a curved surface that passes through the whole dentition is required; in this paper, this curved surface is referred to as the panoramic curved surface. In calculating the position of that surface in 3D data, a reference dental arch curve plays a very critical role. This curve is analogous to the motion trajectory of the source and detector in conventional panoramic radiograph equipment. The method in this paper generates this curved surface and the single-slice panoramic radiograph from dental CBCT data by the following step: (1) creating the dental arch curve in the maximum intensity projection (MIP) of 3D CBCT data; (2) generating the serial sagittal sections of the teeth based on that curve, and extracting the 3D panoramic curved surface from sections; (3) synthesizing the panoramic radiograph by developing the 3D curved surface. Fig 1 shows the work flow of the proposed method. In the rest of this section, we describe the steps more precisely.

### 2.1. Dental arch curve creation

**Fig 2:**
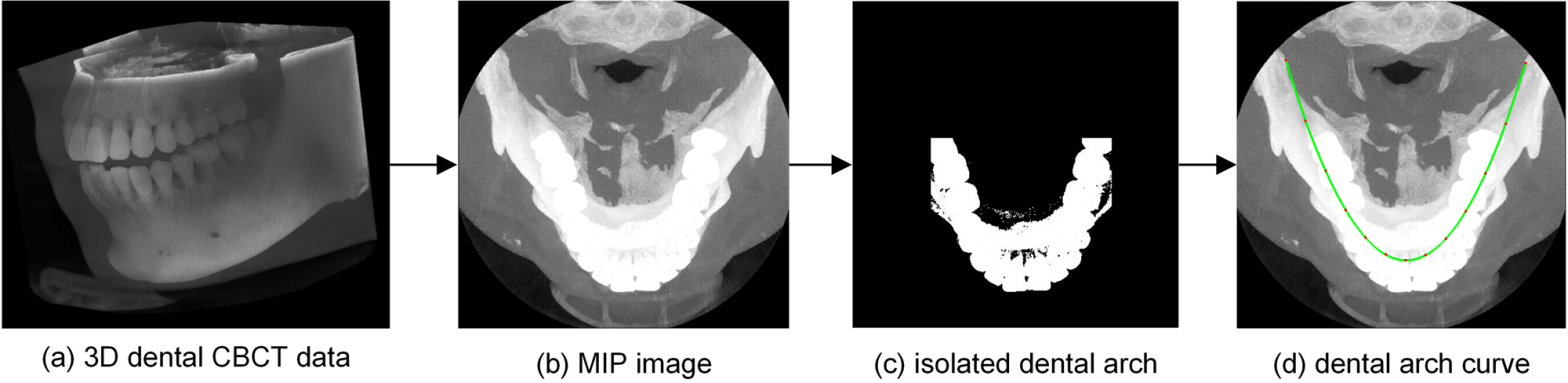
Visualization of the dental arch curve creation applied to a 3D dental CBCT data.

The sub-steps of the dental arch curve creation are shown in Fig 2. Fig 2(a) shows the volume rendering result of the original CBCT dataset, with voxel dimensions of 492×492×303 and a 0.25mm resolution. This dataset was obtained using the dental CBCT imaging system ZCB100 (Shenzhen ZhongKe TianYue Technology Co., Ltd.) at 110 kV and 15 mAs, with a 12.3-cm FOV (field of view). Images from this dataset are uniformly displayed using a window width of 5211 HU and a window level of 1605 HU.

To describe the arch form of the whole dentition, the position of teeth in the horizontal slices is required. Because enamel is the densest tissue in the body, we can produce an MIP image of horizontal slices in the 3D data to create the dental arch curve [16]. This MIP image, which is shown in Fig 2(b), is computed by maintaining the maximum gray value along a ray passing through the same location in all slices.

Then, we use image segmentation methods to isolate the dental arch from the MIP image. Because enamel density is recorded in the form of gray values, we can process this MIP image to isolate teeth based on the gray values. However, the intensities of the teeth and jaws can be very similar in some CBCT datasets. Consequently, the bone area, which includes the dental arch and jaws, is identified by k-means++ clustering; this method automatically provides a gray threshold based on the number of classes *k* [19–21]. As the form of the dental arch is known to be narrower than that of the jaws, a spatial quadrangle including only teeth and no jaws can be established. Specifically, we calculate the centroid point of the bony area and a quadrangle region that covers a certain proportion *p* of pixels in this area. Now, the dental arch can be completely isolated by combining the gray threshold with the spatial threshold. An isolated dental arch, with empirical parameters *k* = 5 and *p* = 75%, is shown in Fig 2(c).

Subsequently, the dental arch curve is created based on the isolated dental arch. AlHarbi et al. found that Hermite cubic spline functions accurately describe irregularly shaped dental arches; however, these functions require control points to be established in advance [22]. To automatically choose the control points, a least squares fitting (LSF) algorithm is used for fitting a parabola based on the coordinates of all the pixel points in the isolated dental arch. The middle point is the apex of the parabola, and the other points are symmetrically distributed on both sides of the apex. The final curve is then acquired by calculating the Hermite cubic spline functions with these control points. This dental arch curve is represented by a green line in Fig 2(d). It should be noted that this final curve follows the average shape of the maxillary and mandibular arches.

### 2.2. Panoramic curved surface extraction

**Fig 3:**
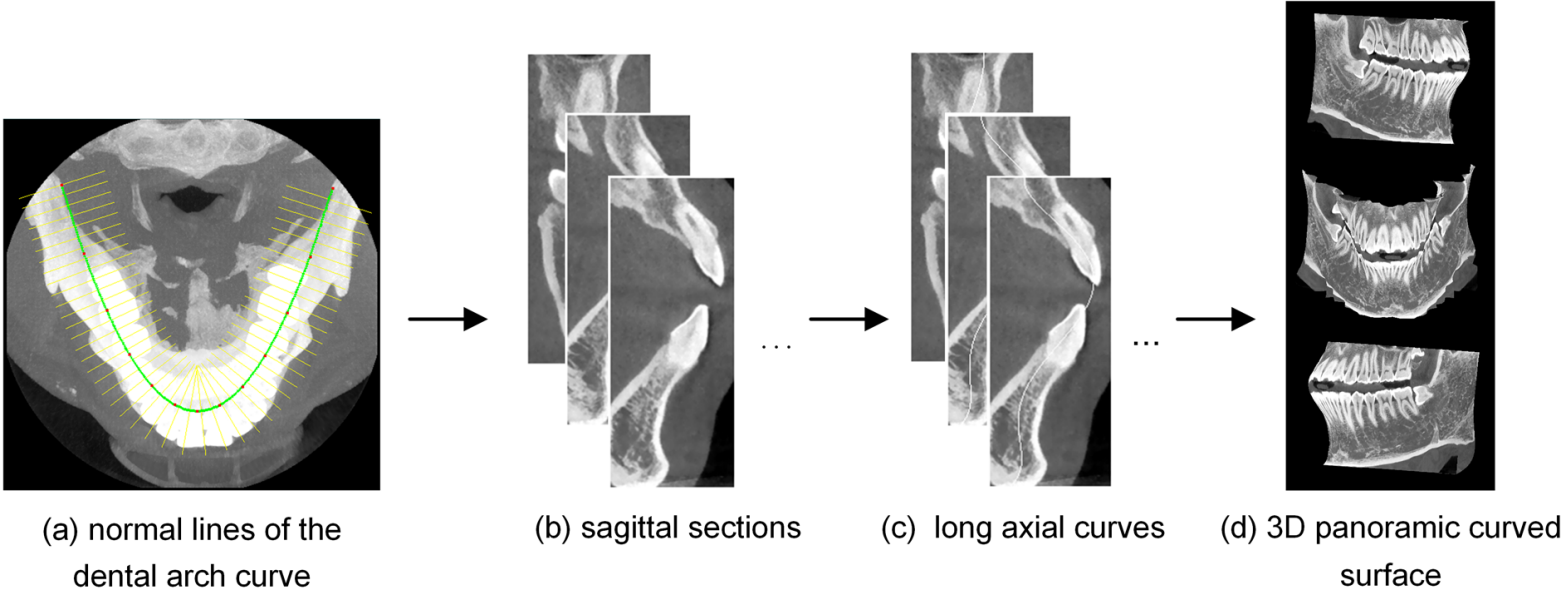
Visualization of the panoramic curved surface extraction applied to a 3D dental CBCT data.

To extract the panoramic curved surface, curves following the shape of the long axes of the upper and lower teeth are created, passing through the upper and lower teeth. These curves are referred to as the long axial curves in this paper, and they can be acquired from sagittal sections perpendicular to the dental arch curve. The major stages of the panoramic curved surface extraction are shown in Fig 3.

The derivative of the Hermite cubic splines can first be used to obtain all the lines perpendicular to the dental arch curve. Fig 3(a) shows one in every ten perpendicular lines for better visualization.

Then, the perpendicular lines are used on all slices to create serial sagittal sections of teeth. Fig 3(b) presents three representative examples of these sections.

To fit the long axial curve, teeth are identified in each section image by a gray threshold method. According to the coordinates of pixel points in the teeth, the long axial curve is generated through quintic curve fitting by LSF. Some corresponding curves are shown in Fig 3(c). By processing all the sections in this way, we can extract all the long axial curves.

All the pixels the serial curves pass through are discrete samples of the actual panoramic curved surface. The panoramic curved surface is extracted using triangle strips indexed from these curves. Three different observations of this surface drawn by OpenGL are shown in Fig 3(d).

### 2.3. Panoramic radiograph synthesis

Panoramic radiograph synthesis can be realized by developing the 3D panoramic curved surface. As the curved surface is composed of serial long axial curves, these curves just need to be successively projected onto straight lines.

Samples of all the long axial curves in a transverse section can be jointed as a piecewise linear curve. We initially straighten the piecewise curve according to the arc length rule:

$$w_{i}=\sum_{j=0}^{i}L_{j},$$ (1)

where *L*_*j*_ represents the length of the *j*th line segment, and *w*_*i*_ denotes the abscissa value at point *i* in the straight line. Then, each long axial curve is projected along the vertical coordinates as follows:

$$h_{j}=z_{j},$$ (2)

where *z*_*j*_ is the vertical value at the sample point *j* in this curve. It should be noted that (*w*_*i*_, *h*_*j*_) is the coordinate of the point *j* in the *i*th long axial curve on the plane.

The single-slice panoramic radiograph is synthesized by straightening all the axial curves using Eqs (1) and (2). The resulting image has a height equal to the number of slices in the CBCT data and a width equal to the number of pixels that the piecewise linear curve passes through.

We can also synthesize a thickened panoramic radiograph by increasing the slice thickness along the normal of the 3D panoramic curved surface. The normal computation method for the surface is available in the PCL [23].

### 2.4. Implementation steps of the proposed method

The proposed method is summarized as follows:

Step 1. Create the dental arch curve:

1. Compute the MIP image of horizontal slices in 3D dental CBCT data (Fig 2(a));
2. Isolate the dental arch (Fig 2(b));
3. Create the dental arch curve (Fig 2(c)).

Step 2. Extract the panoramic curved surface:

1. Calculate lines perpendicular to the dental arch curve (Fig 3(a));
2. Create serial sagittal sections of the teeth (Fig 3(b));
3. Fit long axial curve of the upper and lower teeth on each section image successively (Fig 3(c));
4. Extract the panoramic curved surface with all the long axial curves (Fig 3(d)).

Step 3. Synthesize the panoramic radiograph by developing the panoramic curved surface using Eqs (1) and (2).

## Results

In this section, the performance of the proposed method is evaluated using four dental CBCT datasets: three open-bite shaped datasets and one closed-bite shaped dataset obtained from Shenzhen ZhongKe TianYue Technology Co., Ltd. This company and our laboratory jointly develop the dental CBCT imaging system ZCB100. The four tested datasets were obtained by scanning our researchers using this device and were de-identified. The details of these datasets are listed in Table 1.

**Table 1:** Datasets used to test methods.

Dataset	X-ray source	Detector	kV	mAs	FOV (cm)	Voxel size (mm)	Resolution
Dataset 1	IMD	Varian 2520	110	15	12.3	0.25	492×492×303
Dataset 2	IMD	Varian 2520	110	10	15.36	0.3	512×512×244
Dataset 3	IMD	Varian 2520	110	10	15.36	0.3	512×512×230
Dataset 4	IMD	Varian 2520	110	10	15.36	0.3	512×512×273

### 3.1. Experimental conditions

All experiments were performed on a PC equipped with a 2.30-GHz Intel Xeon E5-2630 dual-core CPU with 16 GB of system memory, and an NVIDIA Quadro K5000 model with 4 GB of video memory. Our method was implemented in the C++ programming language with OpenCV, OpenGL and the Point Cloud Library (PCL) [23].

### 3.2. Quality evaluation of the panoramic radiographs

#### 3.2.1. Open-bite shaped dental CBCT dataset 1

In this experiment, we validated the proposed method and the cylinder method using open-bite shaped dental CBCT dataset 1 described in the Materials and Methods section.

**Fig 4:**
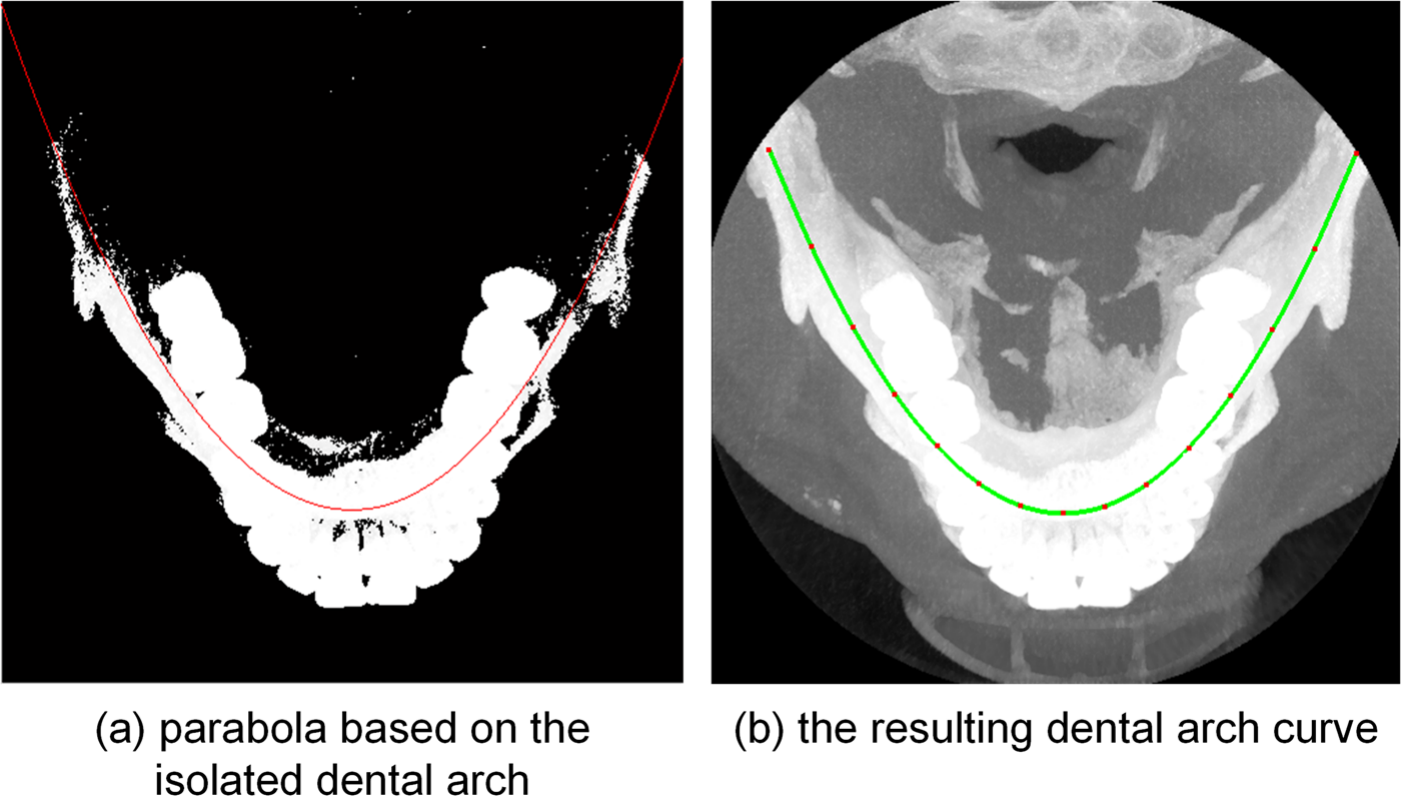
Fitting parabola and dental arch curve based on isolated dental arch without spatial threshold.

Aside from the isolated dental arch shown in Fig 2(c), we tested the dental arch that was isolated without a spatial threshold (i.e., location information). Fig 4 shows the fitting parabola and dental arch curve based on the isolated dental arch. The dental arch isolated with only a gray threshold is shown in Fig 4(a). The red line shown in Fig 4(a) is the fitting parabola. Fig 4(b) shows the resulting dental arch curve added to the MIP image. The final curve was observed to follow the shape of the jaws but not the dentition.

**Fig 5:**
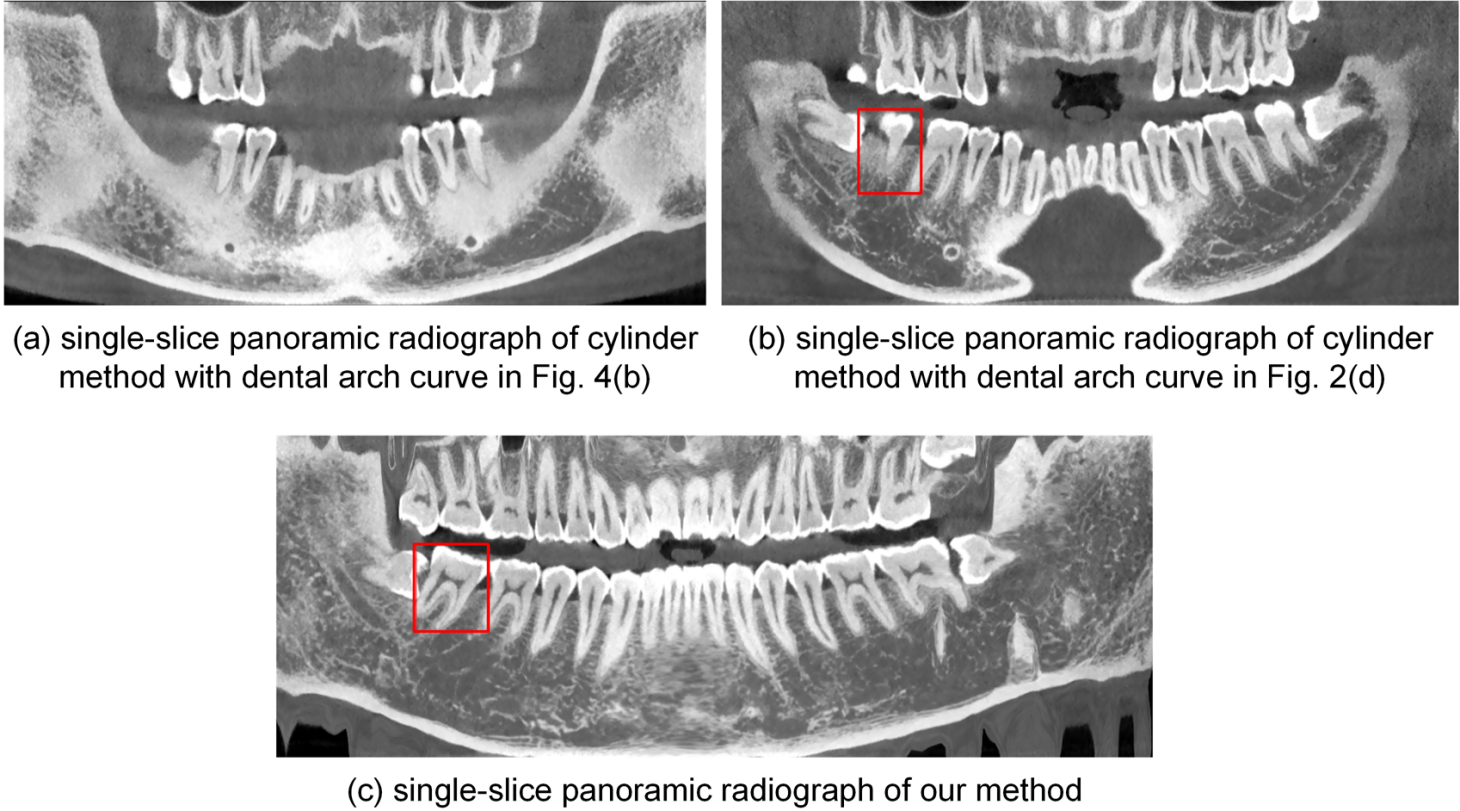
Single-slice panoramic radiographs by different methods from dataset 1.

Single-slice panoramic radiographs synthesized from the open-bite shaped dental CBCT data using the cylinder method and the proposed method are shown in Fig 5 (window width, 5211 HU; window level, 1605 HU). The panoramic radiograph shown in Fig 5(a) was generated by the cylinder method based on the dental arch curve shown in Fig 4(b). Fig 5(b) displays the single-slice panoramic radiograph generated by the cylinder method based on the dental arch curve shown in Fig 2(d). These two single-slice images fail to show some incisors and molars. Fig 5(c) presents the single-slice panoramic radiograph generated by our method, with the developed curved surface shown in Fig 3(d). This experiment with our method used one in every ten normal lines; thus, we processed 73 section images, each of which was 100×303 in size. In each section image, we used the k-means++ (*k* = 4) clustering method to segment the teeth and we fitted a quintic curve to describe the long axes of the upper and lower teeth. The final single-slice image completely shows the whole dentition.

**Fig 6:**
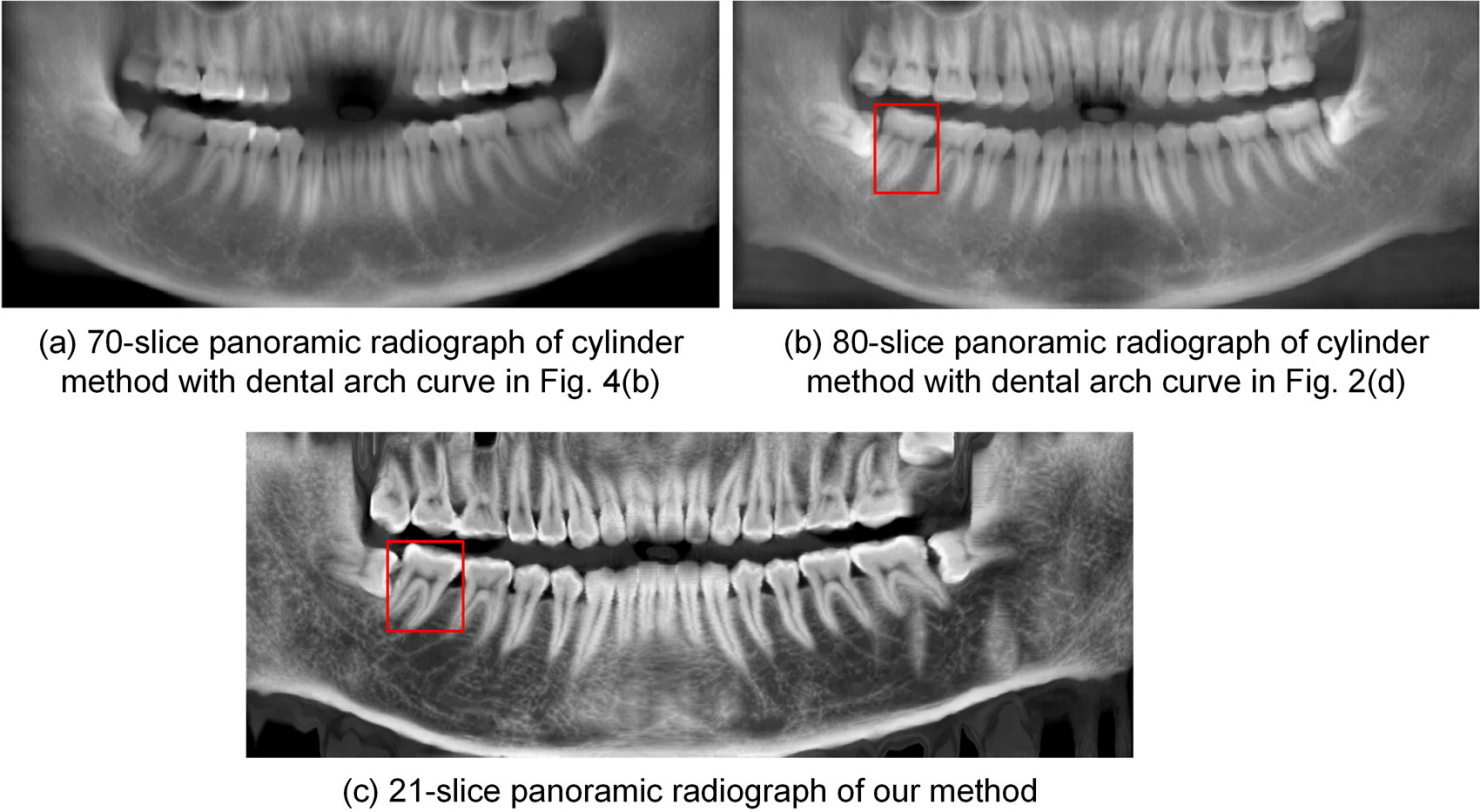
Thickened panoramic radiographs by different methods from dataset 1.

Fig 6 shows the thickened panoramic radiographs generated from the closed-bite shaped dental CBCT data by the cylinder method and the proposed method. Fig 6(a) is an enhanced version of the thickened panoramic radiograph (average image of 70-slice radiographs) produced by the cylinder method with the dental arch curve shown in Fig 4(b). The enhanced method was described in Paris’s paper [24]. The incisors are partially missing in this thickened image. Fig 6(b) provides an enhanced version of the 80-slice panoramic radiograph generated by the cylinder method according to the dental arch curve shown in Fig 2(d). While this radiograph shows the whole dentition, the image is blurry. Fig 6(c) shows an enhanced version of the 21-slice panoramic radiograph generated by our method, and it clearly shows the whole dentition. In addition, it uses fewer slices than does the cylinder method. The thickened panoramic radiographs show more structures, such as the mandibular nerve canal, than do the single-slice images.

**Fig 7:**
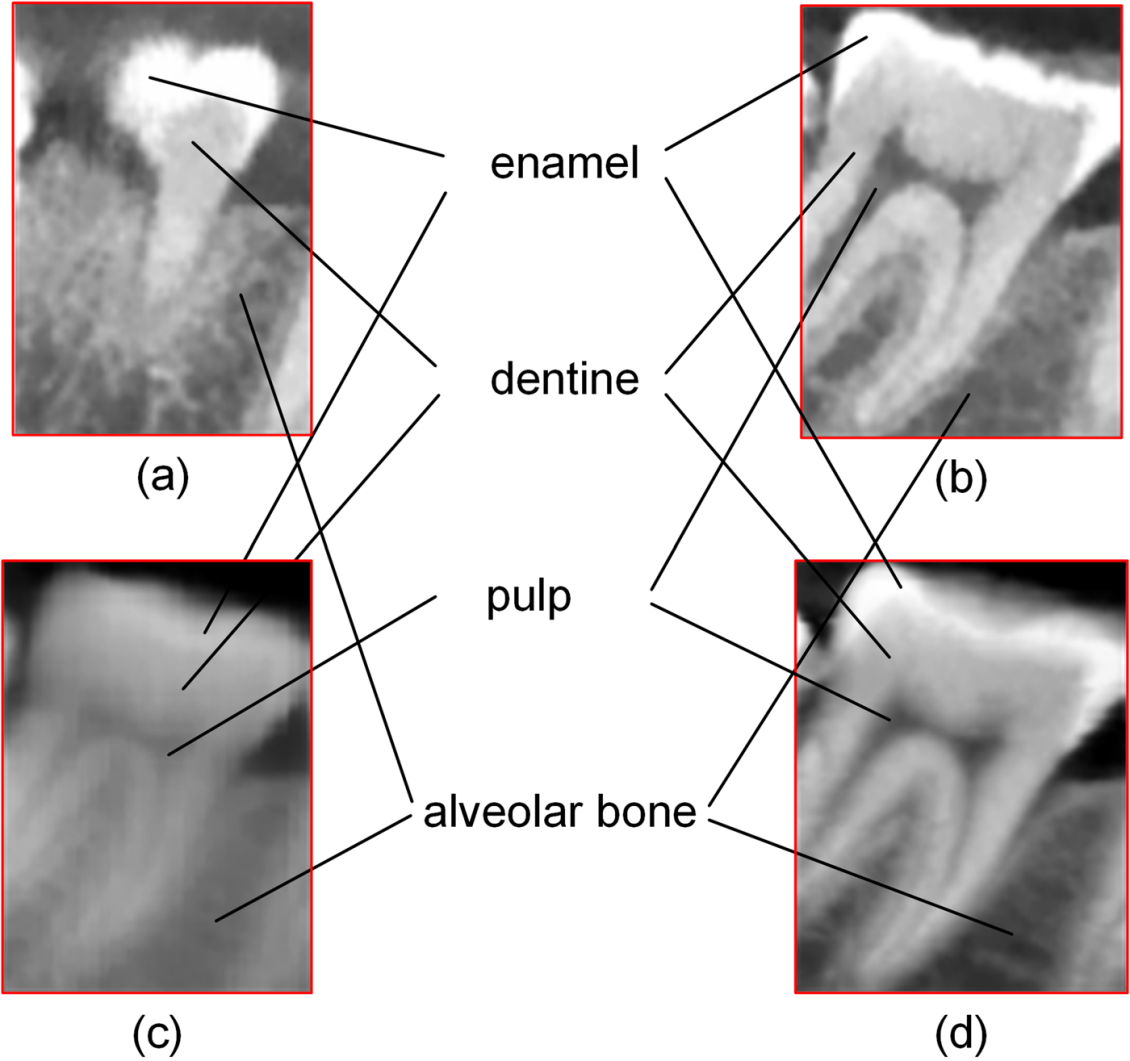
Anatomical structures in panoramic radiographs: (a) single-slice panoramic radiograph by cylinder method, (b) single-slice panoramic radiograph by our method, (c) thickened panoramic radiograph by cylinder method, (d) thickened panoramic radiograph by our method.

Different anatomical structures of selected teeth in various panoramic radiographs are shown in Fig 7. Fig 7(a)–7(d) shows the selected teeth marked with red boxes in Figs 4(a), 4(b) and 5(a), 5(b), respectively.

#### 3.2.2. Closed-bite shaped dental CBCT dataset 2

Panoramic imaging can be implemented in dental practice through several 3D imaging software packages, such as OnDemand3D (Cybermed Inc.), SimPlant (Materialise Inc.), and eXam Vision (KaVo Dental GmbH). In OnDemand3D, practitioners can create the dental arch curve manually or automatically and obtain panoramic radiographs corresponding with this curve. In the trial version of Simplant, the dental arch curve can only be produced by manually selecting a slice and choosing control points. While the eXam Vision software can create the curve and panoramic radiographs automatically, the education version we downloaded could not process the particular external datasets tested in this paper. Therefore, we decided to compare the OnDemand3D software with our method. In this experiment, we tested the methods using a closed-bite shaped dental CBCT dataset. Images from this dataset are displayed with the following uniform window setting: window width, 18730 HU; window level, 9365 HU.

**Fig 8:**
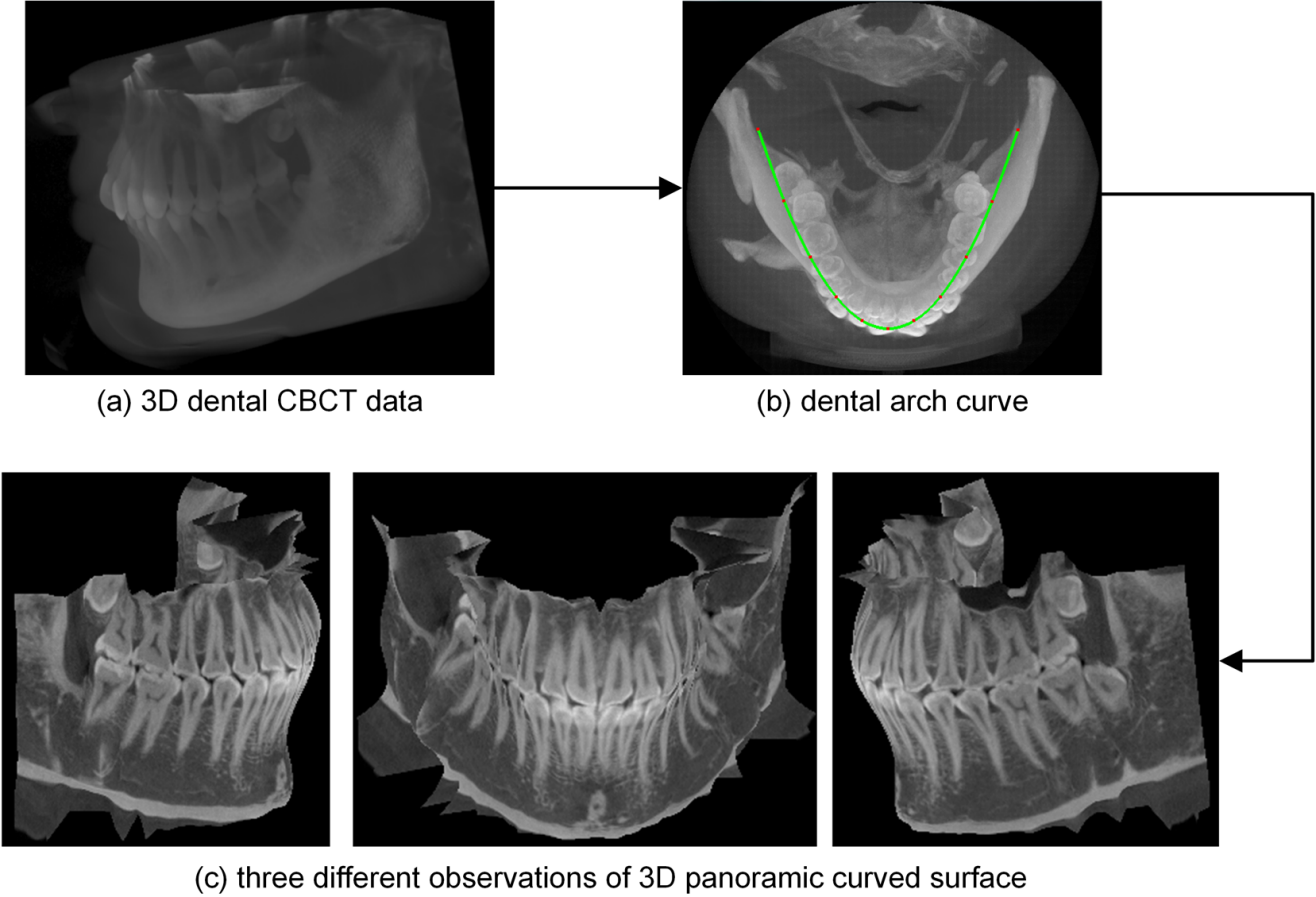
Results of our method from the closed-bite shaped dental CBCT dataset 2.

Fig 8 shows the dental arch curve and 3D panoramic curved surface generated by our method from closed-bite shaped dental CBCT dataset 2. In this experiment, one in every ten normal lines was used; thus, we processed 61 section images, each of which was 100×244 in size. In each section image, the authors used k-means++ (*k* = 2) clustering method to segment teeth and the other necessary parameters were the same as those used in experiment 1. Fig 8(a) shows the volume rendering result of the original data. The dental arch curve added to the MIP image of the slices is shown in Fig 8(b). Fig 8(c) shows three different observations of the 3D panoramic curved surface; the whole dentition is clearly shown in the final 3D surface.

**Fig 9:**
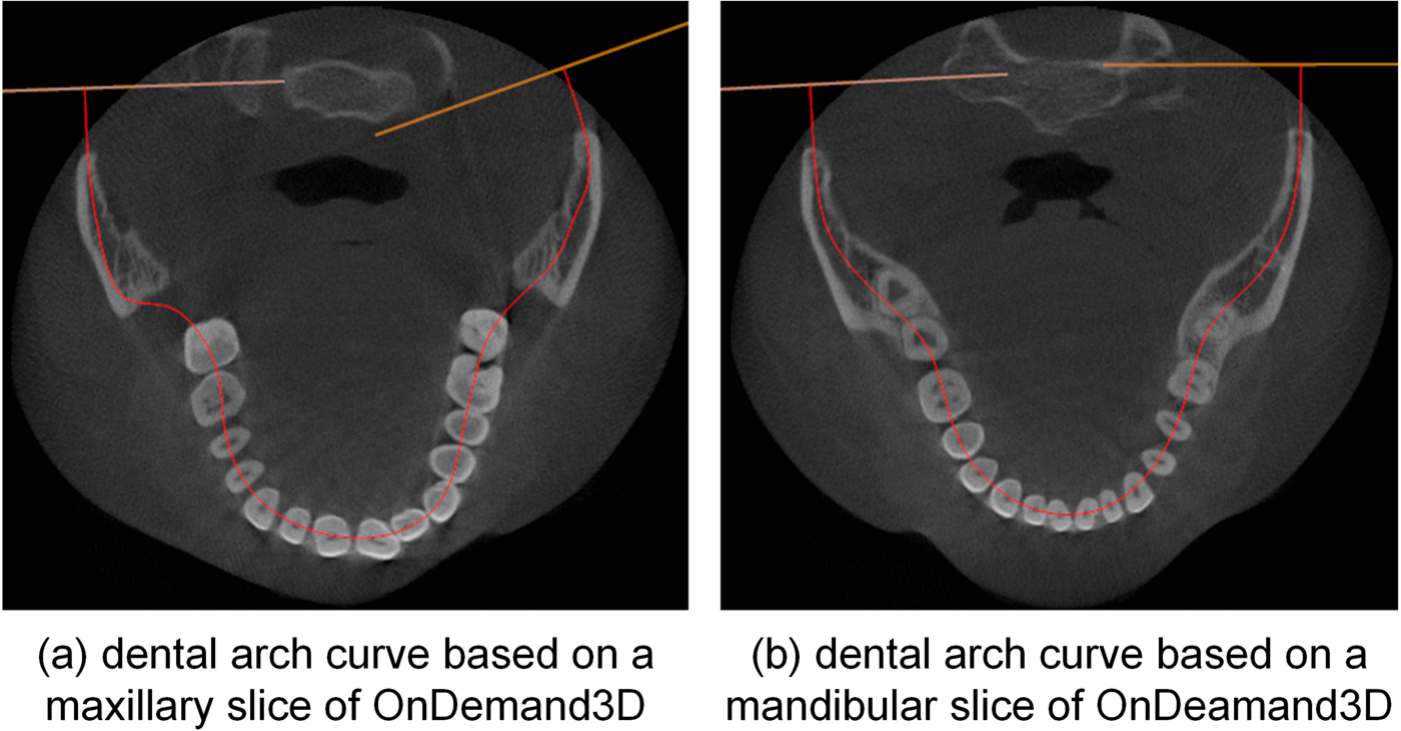
Automatic dental arch curves of OnDemand3D.

The automatic dental arch curves created from closed-bite shaped CBCT dataset 2 by OnDemand3D are shown in Fig 9. The red curves shown in Fig 9(a) and 9(b) are fitted based on a maxillary slice and a mandibular slice, respectively.

**Fig 10:**
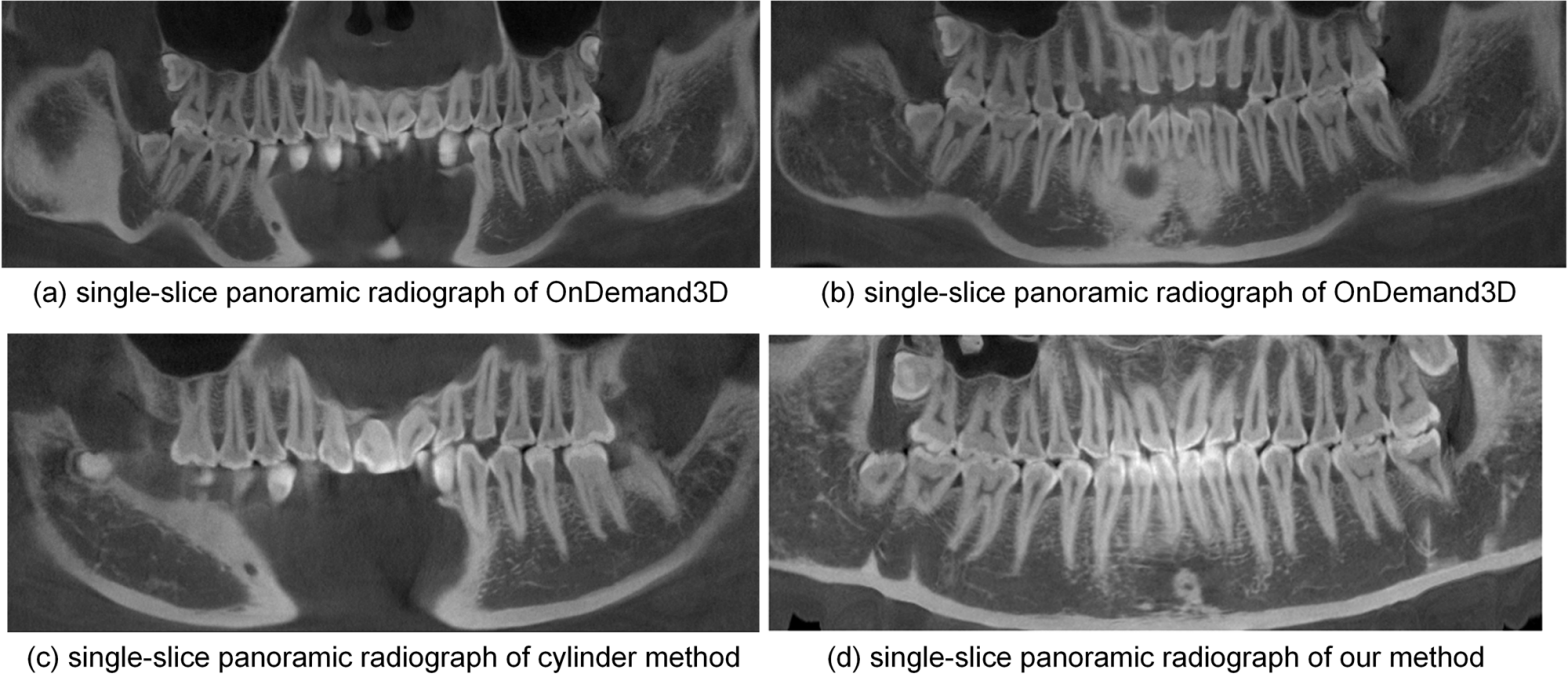
Single-slice panoramic radiographs by different methods from dataset 2.

Single-slice panoramic radiographs synthesized from closed-bite shaped dental CBCT dataset 2 by the different methods are shown in Fig 10. Fig 10(a) and 10(b) are based on the curves shown in Fig 9(a) and 9(b), respectively. The image based on the maxillary curve fails to show some incisors and molars. While the image shown in Fig 10(b), based on the mandibular curve, is a higher quality image than (a), some teeth are still missing. Fig 10(c) and 10(d) are based on the same dental arch curve (Fig 8(b)). The image generated by the cylinder method (Fig 10(c)) fails to show some incisors and molars, so it cannot provide an accurate number of teeth. In the single-slice image produced by our method (Fig 10(d)), the number of teeth can be identified quickly and accurately, demonstrating that our method performs better than the others in rendering dentition and anatomic structures.

**Fig 11:**
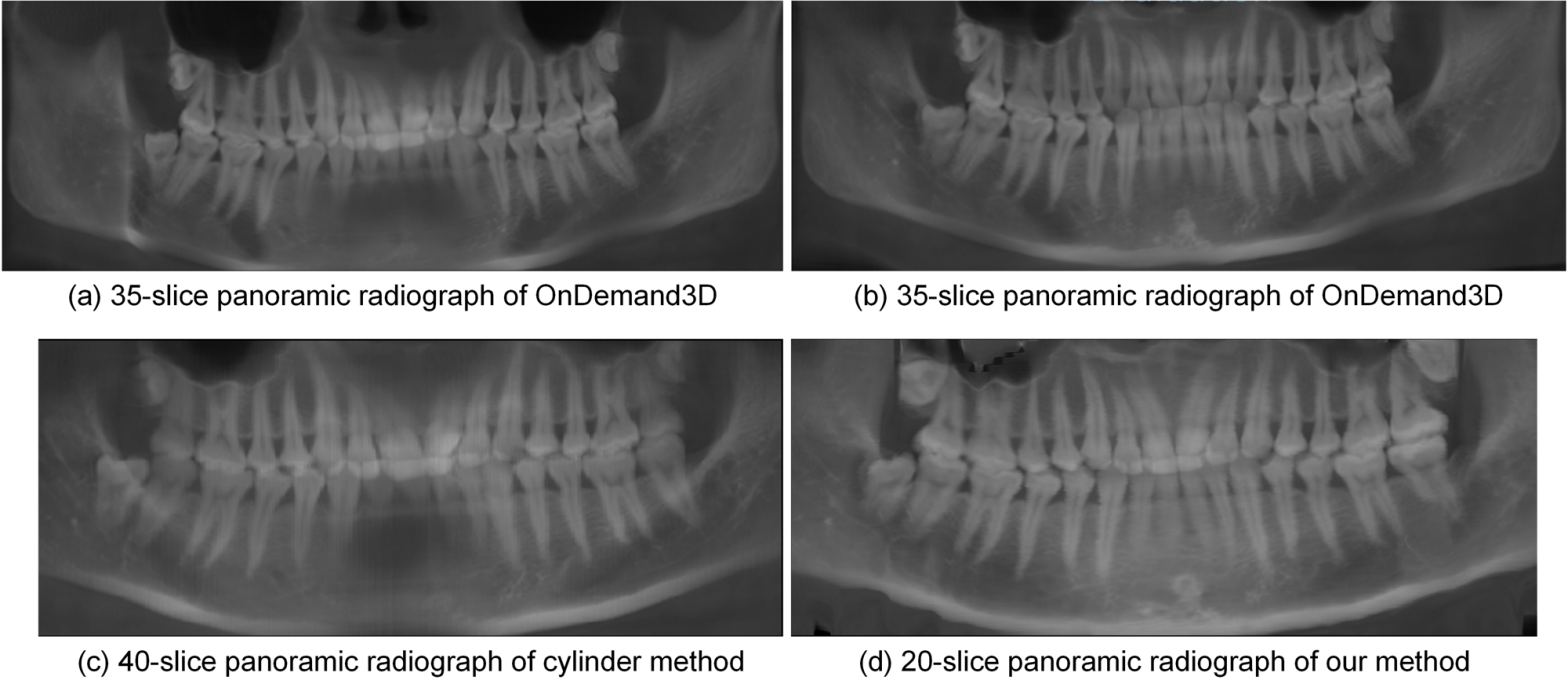
Thickened panoramic radiographs by different methods from dataset 2.

Fig 11 shows thickened panoramic radiographs from closed-bite shaped dental CBCT dataset 2 generated by different methods. Fig 11(a) is based on the red curve shown in Fig 9(a) and 9(b) is based on the red curve shown in Fig 9(b), 9(c) and 9(d) are based on the curve shown in Fig 8(b). Fig 11(c) is the thickened panoramic radiograph (average image of 40-slice radiographs) generated by the cylinder method. Fig 11(a) and 11(c) show teeth with inconsistent clarity, and some incisors are missing. Fig 11(d) shows a 20-slice image thickened by our method. Fig 11(b) and 11(d) show the whole dentition with consistent clarity, and our method required fewer slices than the other methods to produce the image shown in Fig 11(d).

A comparison of the panoramic radiographs generated by applying the different methods to the closed-bite shaped dental CBCT dataset is summarized in Table 2.

**Table 2:** Comparison of panoramic radiographs by different methods on the closed-bite shaped dental CBCT data.

Method	Result	Thickness (slice)	Manual option	Whole dentition
OnDeamand3D	Fig 10(a)	1	Select a slice	No
	Fig 10(b)	1	Select a slice	No
	Fig 11(a)	35	Select a slice	No
	Fig 11(b)	35	Select a slice	Yes
Cylinder	Fig 10(c)	1	No	No
	Fig 11(c)	40	No	No
Proposed method	Fig 10(d)	1	No	Yes
	Fig 11(d)	20	No	Yes

#### 3.2.3. Open-bite shaped dental CBCT dataset 3

In this experiment, we validated the proposed method and the cylinder method using open-bite shaped dental CBCT dataset 3. In this dataset, the parabola was unable to follow the shape of the dental arch.

**Fig 12:**
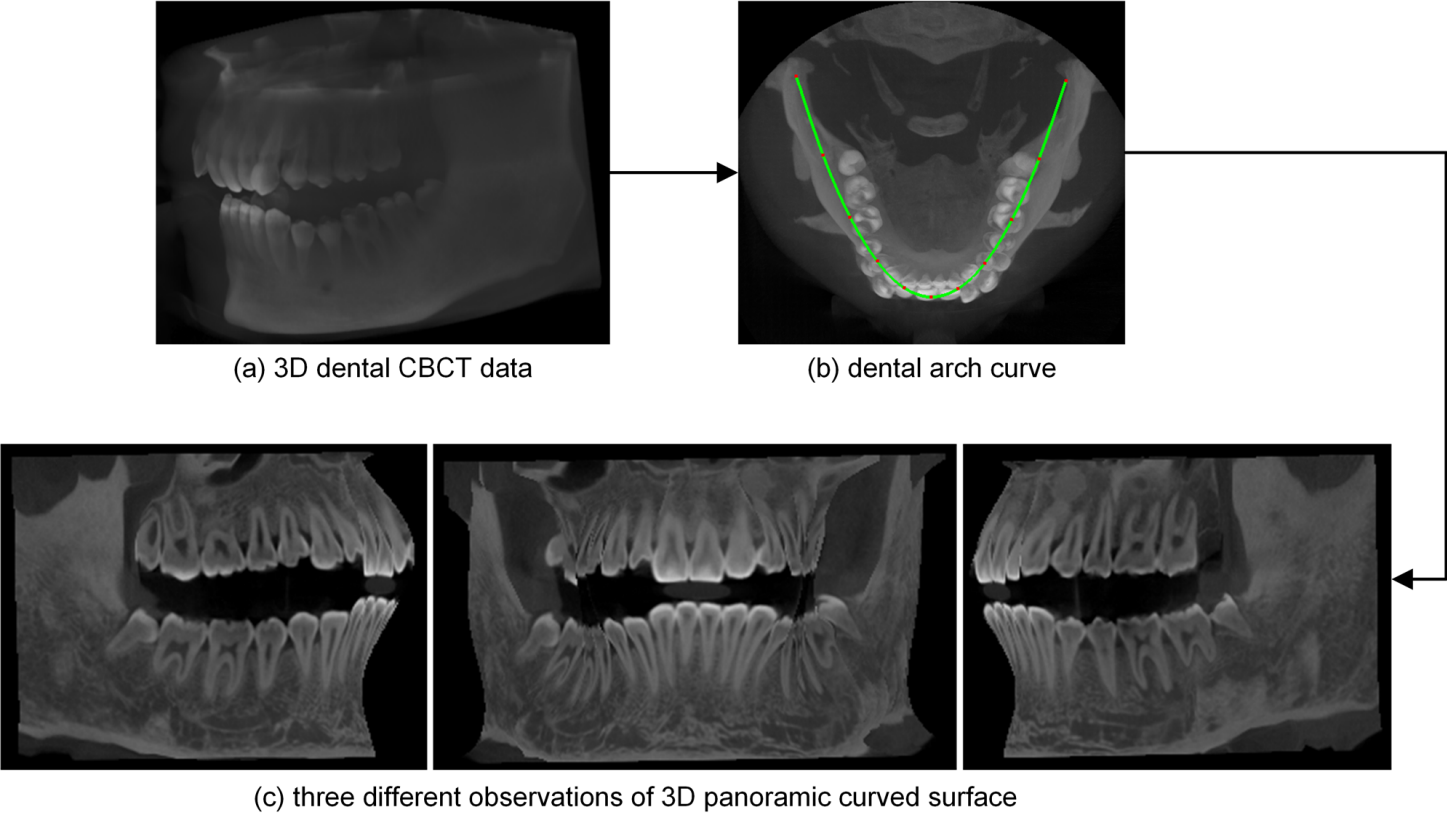
Results of our method from dataset 3.

The dental arch curve and 3D panoramic curved surface generated by our method using dataset 2 are shown in Fig 12 (window width, 16384 HU; window level, 8192 HU). In this experiment, one in every ten normal lines was used; thus, we processed 62 section images, each of which was 80×230 in size. The other necessary parameters were the same as those used in experiment 2. Fig 12(a) shows the volume rendering result of the original data. The dental arch curve added to the MIP image of the slices is shown in Fig 12(b), while Fig 12(c) shows three different observations of the 3D panoramic curved surface. The whole dentition is clearly shown in the final 3D surface.

**Fig 13:**
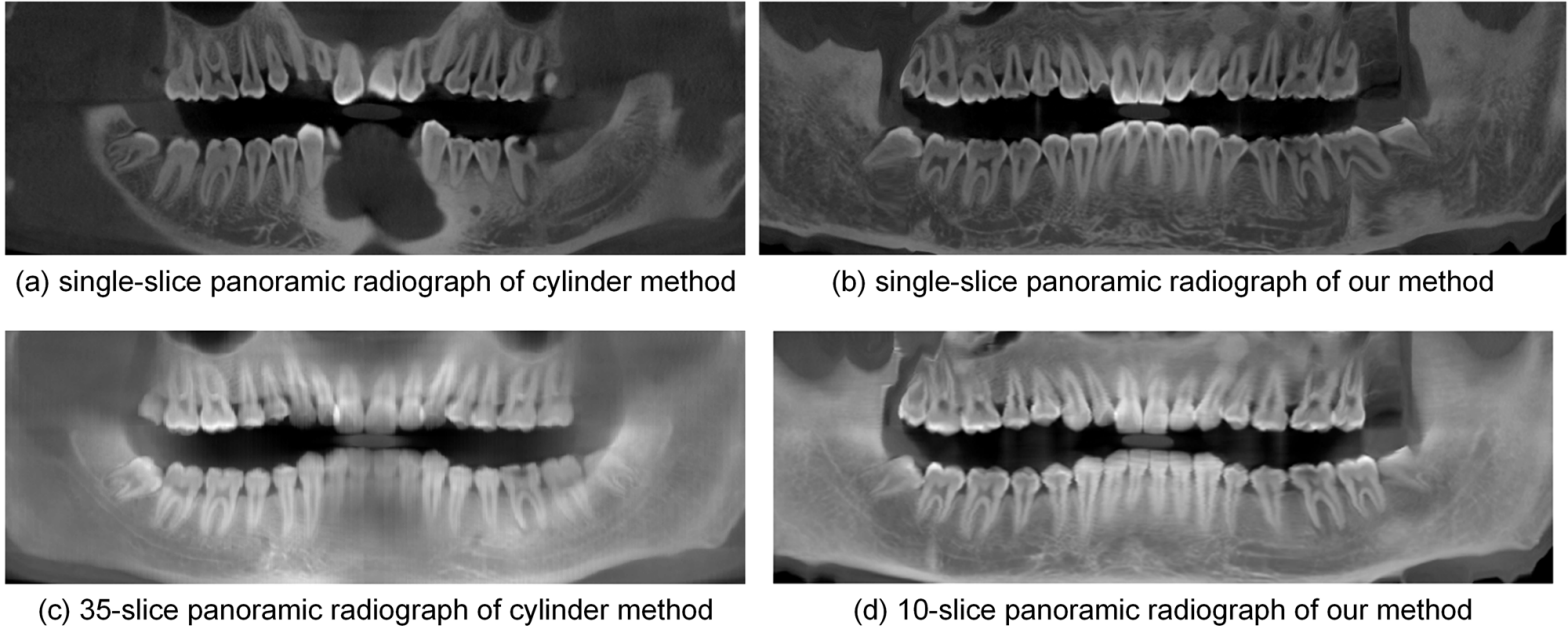
Panoramic radiographs by different methods from dataset 3.

Fig 13 shows panoramic radiographs synthesized from dataset 3 by the cylinder method and the proposed method. Fig 13(a) and 13(b) are displayed with the following window settings: window width, 16384 HU; window level, 8192 HU. Fig 13(c) and 13(d) are displayed with the following window settings: window width, 11655 HU; window level, 5828 HU. These images are based on the same dental arch curve (Fig 12(b)). Fig 13(a) is the single-slice image generated by the cylinder method; it fails to show incisors and molars, so it cannot provide an accurate number of teeth. In the single-slice image generated by our method (Fig 13(b)), the number of teeth can be identified quickly and accurately. Fig 13(c) shows the thickened panoramic radiograph (average image of 35-slice radiographs) generated by the cylinder method. It shows teeth with inconsistent clarity and misses some molars. Fig 13(d) shows an image thickened by 10 pixels by our method. This image exhibits the whole dentition with consistent clarity. In addition, the mandibular curve canals are visible in the thickened results.

#### 3.2.4. Open-bite shaped dental CBCT dataset 4

In this experiment, we validated the proposed method and the cylinder method using open-bite shaped dental CBCT dataset 4that is with dentition defect, enamel defects and metallic implant.

**Fig 14:**
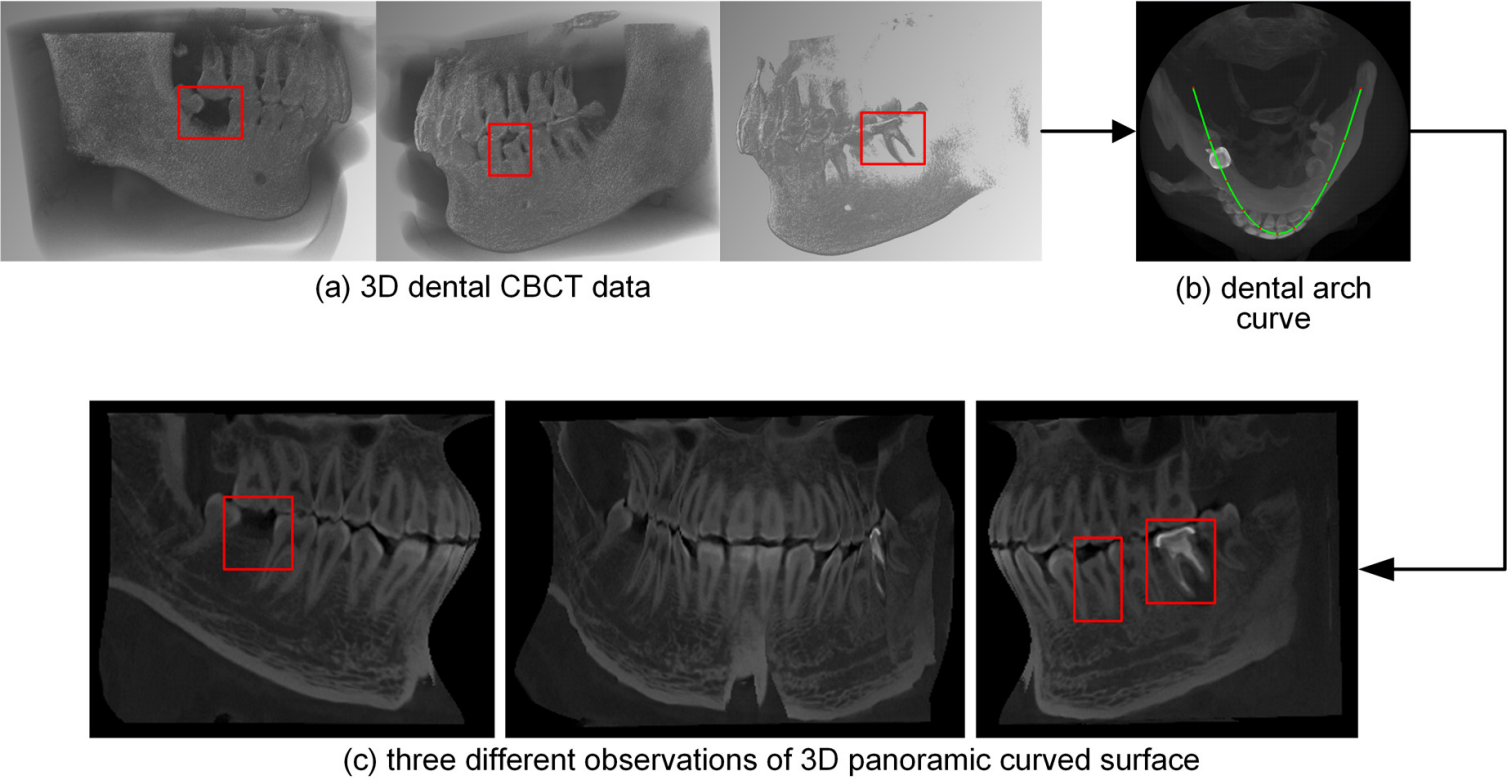
Results of our method from dataset 4.

Fig 14 (window width, 16357 HU; window level, 8178 HU) shows the dental arch curve and 3D panoramic curved surface generated by our method from dataset 4. In this experiment, one in every ten normal lines was used; thus, we processed 62 section images, each of which was 67×273 in size. The other necessary parameters were the same as in experiment 2. Fig 14(a) shows the volume rendering result of the original data. The dental arch curve added to the MIP image of the slices is shown in Fig 14(b). Fig 14(c) shows three different observations of the 3D panoramic curved surface. The tooth loss, enamel defects and metallic implant are marked with red boxes in these images.

**Fig 15:**
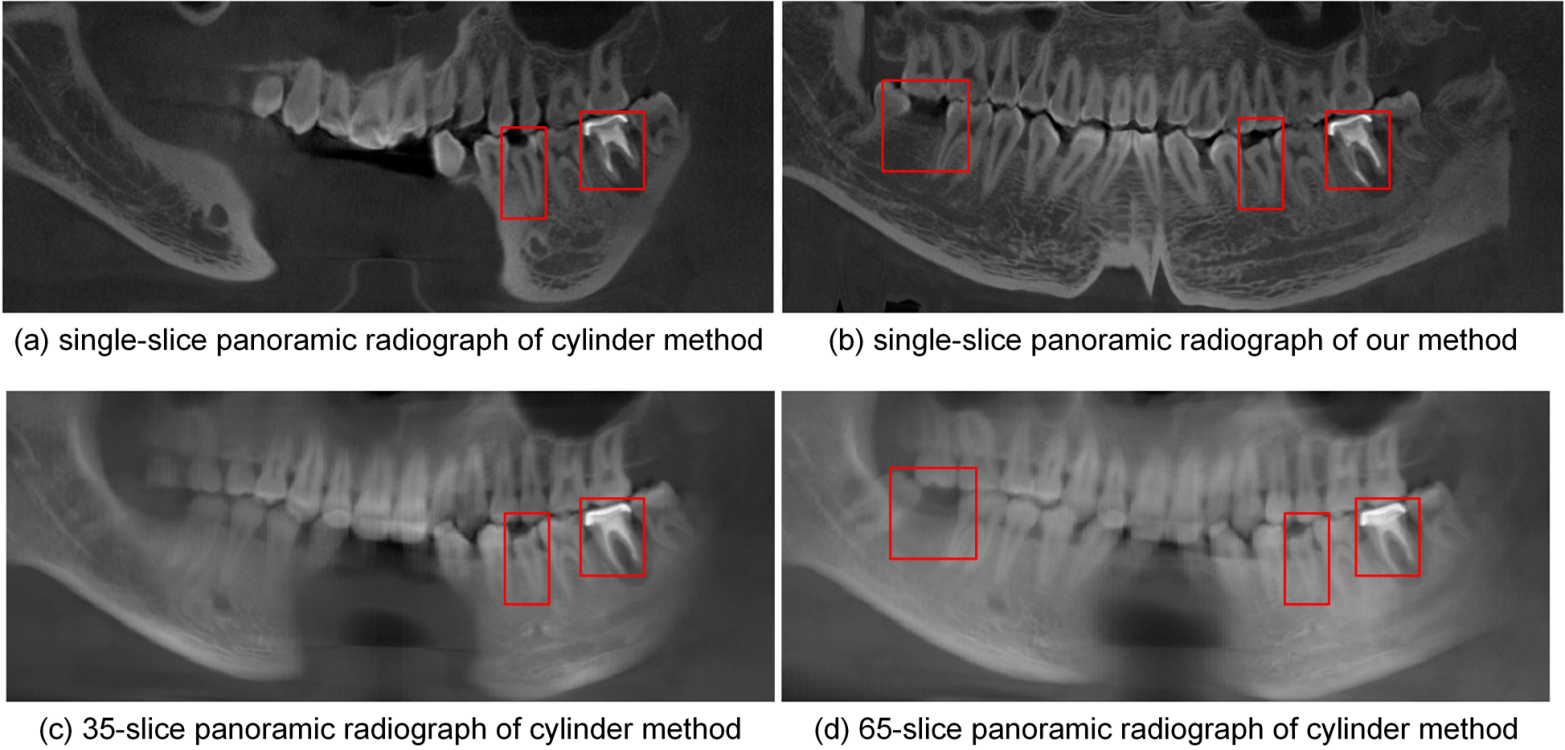
Panoramic radiographs of different methods from dataset 4.

Panoramic radiographs synthesized from dataset 4 by the different methods are shown in Fig 15. The resulting images are based on the same dental arch curve (Fig 14(b)). Fig 15(a) and 15(b) are displayed with the following window settings: window width, 16357 HU; window level, 8178 HU. Fig 15(a) fails to show some incisors and molars, in particular the tooth loss. In the single-slice image resulted by our method (Fig 15(b)), the tooth loss, enamel defects and metallic implant can be identified quickly and accurately. Fig 15(c) and 15(d) are displayed with the following window settings: window width, 10340 HU; window level, 5170 HU. While more dental structures were shown in Fig 15(c) (average image of 35-slice radiographs) than (a), the tooth loss still cannot be identified. Increasing the slice thickness to 65 (Fig 15(d)) finally shows the structure of tooth loss.

### 3.3. Time evaluation

The time cost of the proposed method was also evaluated at each step, and the results from these datasets are listed in Table 3. According to this table, the time costs of the whole process applied to the tested datasets are all less than 7 seconds. We also analyzed the time cost of the other methods applied to the closed-bite shaped dental CBCT data, and the results are listed in Table 4. The manual and automatic OnDemand3D processing methods used to create the dental arch curve required more than 2 and 7 seconds, respectively. The classic cylinder method required 2.09100 seconds.

**Table 3:** The time cost of the proposed method applied to these datasets.

Dataset	Time (seconds)			
	Step 1	Step 2	Step 3	The whole time
Dataset 1 (size: 492×492×303)	0.84862200	5.79090000	0.00770953	6.68018000
Dataset 2 (size: 512×512×244)	2.10585000	3.7295000	0.00981153	5.84596000
Dataset 3 (size: 512×512×230)	2.11387000	3.90158000	0.00880467	5.9607000
Dataset 4 (size: 512×512×273)	1.99936000	4.26875000	0.00856368	6.28312000

**Table 4:** The time cost of different methods in experiment 2.

Method	Process	Time (seconds)
OnDemand3D	manually select a slice; manually choose control points (>7 points); the rest of the processes is automatic	>7.00000
OnDemand3D	manually select a slice; the rest of the processes is automatic	>2.00000
Cylinder method	Step 1; cylinder method; all the processes is automatic	2.09100
Proposed method	Step 1; step 2; step 3; all the processes is automatic	5.84596

## Discussion

This paper presents a method for directly observing the whole dentition by synthesizing panoramic radiographs from dental CBCT data without superimposing other structures. There are two major challenges for this type of synthesis: (i) how to find a dental arch curve for used in the synthesis of the panoramic radiograph; and (ii) how to extract the 3D CBCT data into a panoramic radiograph. Comparing the panoramic radiographs shown in Figs 5(a) with 5(b), 6(a) with 6(b), 10(a) with 10(b), and 11(a) with 11(b), we can conclude that resulting panoramic radiographs depend on the given dental arch curve.

### 4.1 Dental arch curve creation

Ordinarily, the dental arch curve is created on either a single horizontal slice or the MIP of all slices. As shown in Fig 9, the dental arches of the maxilla and mandible are very different. A curve in an individual slice cannot describe the dental arch form of the whole dentition well because it is only valid in a single slice. The dental arch curve shown in Fig 4(b) was created using the MIP; however, some anterior teeth and maxillary molars are still poorly represented.

In this paper, we also created the dental arch curve based on the MIP image. According to the gray and spatial information recorded in the MIP image, we isolated the dental arch (step 1(b)), and then fitted the dental arch curve with parabolic and cubic spline functions. A comparison between Figs 2(d) and 4(b) shows that adding the constraint of the spatial information reduces the influence of the jaws on the resulting dental arch curve. Hence, the final dental arch curve shown in Fig 2(d) passes through the whole dentition more accurately than do the curve shown in Fig 4(b).

**Fig 16:**
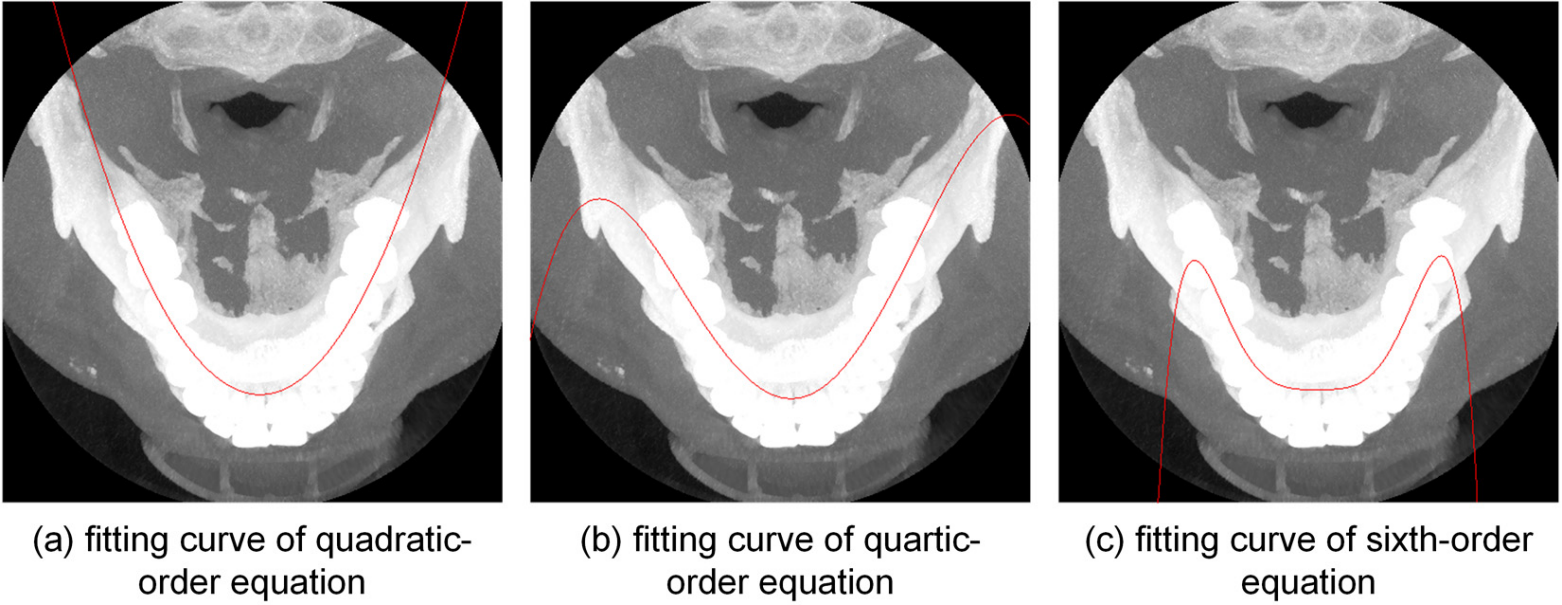
Different fitting curves add to the MIP image.

The dental arch has been described in many laconic mathematical formulas, such as catenary curves [25], beta functions [26], cubic splines [27, 28], and other polynomial functions [29]. Among them, the catenary curves and beta functions require the location of some teeth, such as the first and second molars, which is difficult to acquire automatically. While a cubic spline requires control points in advance, which can be given manually or automatically, polynomial functions can be fitted automatically. We tested three different polynomial curves based on the isolated dental arch, and the results are shown in Fig 16. These images show that quartic and sextic curves are incorrect at the ends of the dental arch; therefore, the quadratic curve was a better choice for this method. After automatically choosing control points from a quadratic curve, i.e., a parabola, we calculated the Hermite cubic spline functions for describing the dental arch to allow the curve to be adjusted by moving the control points.

### 4.2 Panoramic radiograph synthesis

After generating the dental arch curve, we need to confirm the location of structures in the 3D dental CBCT data, which should be displayed in the panoramic radiograph.

As discussed in the Introduction section, many studies extract data using a cylinder based on the dental arch curve. However, because there is an angle between the long axis of the teeth and the vertical direction, the single-slice panoramic radiographs produced by this cylinder method fail to show the whole dentition. This effect is demonstrated in Figs 5(a), 5(b) and 10(a), 10(c) and 13(a) and 15(a). According the results shown in Fig 10(a) and 10(c), we speculate that the OnDemand3D software uses the cylinder method. Single-slice panoramic radiographs are rarely used in dental practice because their failure to show the whole dentition.

In this paper, we use a polynomial surface to describe the whole dentition. We chose a quintic curve to follow the long axial curve of the teeth, and then we produced a panoramic curved surface by indexing all these axial curves. The final panoramic curved surfaces generated from open-bite shaped and closed-shaped dental CBCT datasets are shown in Figs 3(d) and 8(c), respectively. These surfaces show the whole dentition well. Although the parabola was unable to follow the shape of dental arch in dataset 3, the 3D panoramic curved surface (Fig 12(c)) can also describe the whole dentition well. There are dentition defect, enamel defects and metallic implant in dataset 4, and the final 3D surface (Fig 14(c)) presents the actuality of the dentition.

**Fig 17:**
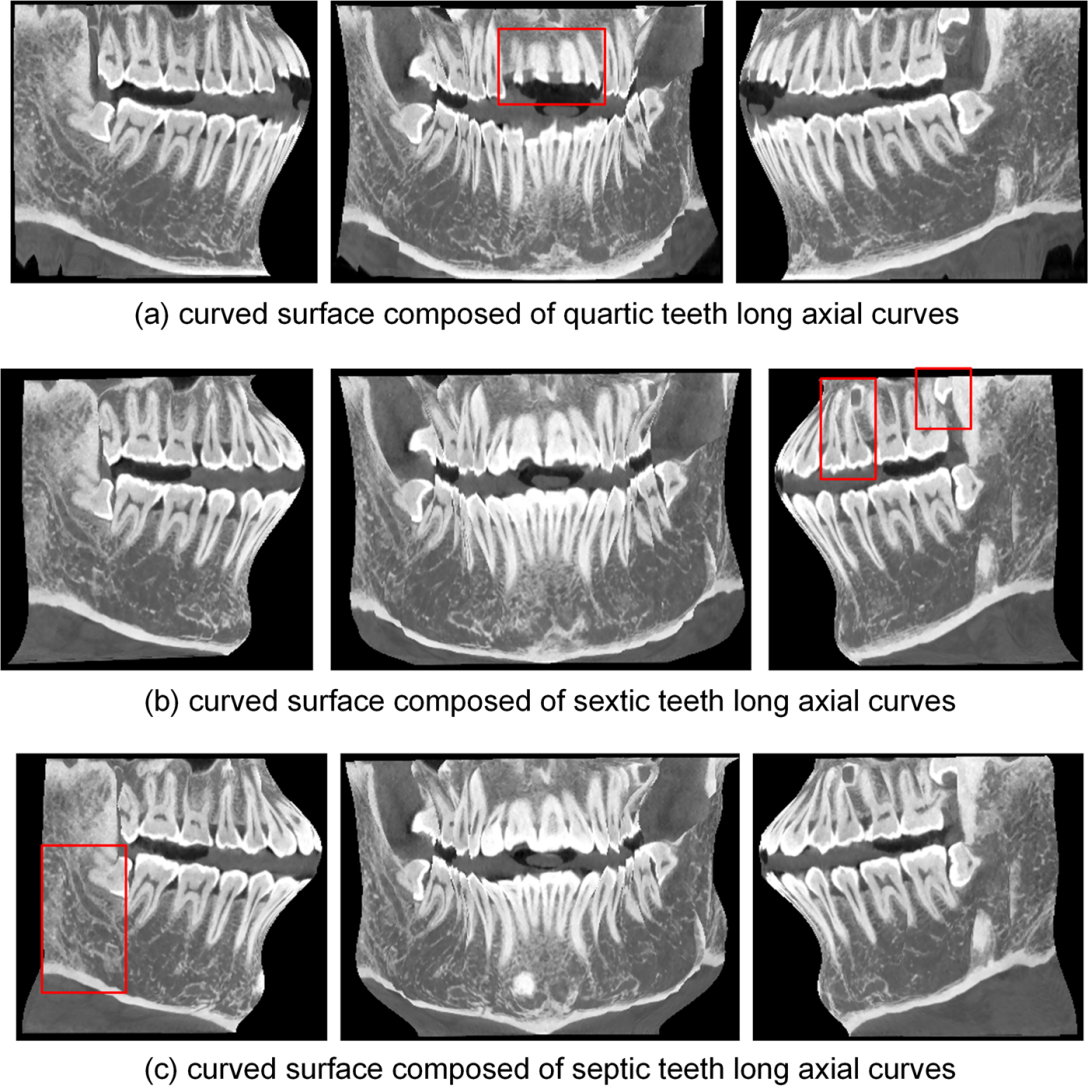
Curved surfaces composed of different long axial curves.

In addition to the quintic curve, we also used three other polynomial curves to describe the long axes of the teeth, and the resulting curved surfaces are shown in Fig 17. Compared with the surface shown in Fig 3(d), the surface shown in Fig 17(a), composed of quartic curves, is missing some incisors. The image shown in Fig 17(b) is similar to that in Fig 3(d) except for some details, such as regions close to the molars. Fig 17(c) shows a surface composed of septic curves that is disordered in some regions; high-order curves result in disorder, which leads to distortions in the final panoramic radiograph. The incorrect areas in Fig 17 are marked with red boxes. These results indicate that the quintic polynomial curve is the better choice for describing the long axes of upper and lower teeth.

As shown in Figs 5(c), 10(d) and 13(b), the single-slice panoramic radiographs generated by our method clearly show the whole dentition. In particular, the Fig 15(b) accurately presents the tooth loss, enamel defects and metallic implant in the original dataset. Hence, a curved surface extracted by our method is better than a cylinder for describing the whole dentition using open-bite shaped and closed-bite shaped dental CBCT datasets. Our method yields more dental information than other methods do using single-slice images, e.g., the number of teeth, which is useful in dental practice.

Currently, thickened panoramic radiographs have been widely used in clinical environments. Thickened panoramic radiographs show more structures than single-slice images, including the mandibular nerve canal. By comparing Figs 6(b) with 6(c), 11(c) with 11(d) and 13(c) with 13(d), the results demonstrate that the cylinder method requires more slices than our method to show the whole dentition, which causes the resulting image to be blurrier. Regarding the closed-bite shaped dental CBCT dataset 2, Fig 11(a) shows the 35-slice panoramic radiograph produced using OnDemand3D. This radiograph is similar to the 40-slice panoramic radiograph generated by cylinder method, shown in Fig 11(c), which supports the conclusion that the OnDemand3D software uses the cylinder method. The results shown in Fig 11(a) and 11(b) support the conclusion that the mandibular curve performs better than the maxillary curve in the cylinder method.

According to clinicians' assessment, panoramic radiographs generated by our method can clearly show dental structures. A comparison of selected teeth in various panoramic radiographs (Fig 7) shows that different anatomical structures, such as enamel, dentine, pulp, and alveolar bone, are rendered more completely and clearly by our method than by the cylinder method. In the experiment 4, it is not until 65-slice thickness that the dentition loss can be shown in thickened result; by contrast, this structure is presented in single-slice result of our method (Fig 15(b)). These observations demonstrate that thinner images generated by our method are helpful for clinicians quickly and accurately identifying information, such as the number of teeth and enamel defects.

The panoramic radiographs generated by our method are useful for observing the number of teeth and enamel defects; this method could also be used in further researches, e.g., for investigating teeth recognition in CBCT data [11–14]. Although the panoramic radiographs generated by our method have slight geometric distortions in the incisors and the ends of the jaws, doctors can observe these structures in 3D CBCT data, volume rendering results or 2D planar reformations. In addition, some jagged artifacts are observed in thickened images because of errors in normal estimation. The attenuation of these geometric distortions and normal errors will be subjects of future research. According to these experiments, the parameter *k* for teeth segmentation is uniform, so the Otsu algorithm can be used in process of automatic segmentation [30, 31].

### 4.3 Time cost

According to Table 3, the single-slice panoramic radiographs automatically yielded by our method required less than 7 seconds. Step 2, extracting the panoramic curved surface, required the majority of this time. As listed in Table 4, practitioners using the OnDemand3D software need to manually select a slice, and then they can either manually choose control points to create an arch curve or automatically create an arch curve. The time cost of the manual options depends on the skill level of the doctor and the oral situation of the patient. These two procedures required more than 7 seconds and 2 seconds. The cylinder method only required approximately 2 seconds, making it the fastest method. Based on the observed image quality and time cost, the proposed method is a viable option for generating panoramic radiographs in clinical dental practices.

## Conclusion

In this paper, we present an automatic method for synthesizing panoramic radiographs from dental CBCT data. This method extracts a 3D panoramic curved surface to describe the whole dentition, and then yields single-slice panoramic radiographs by developing the 3D surface. This method is also capable of synthesizing thickened panoramic radiographs by increasing the slice thickness. We tested this method on both open-bite shaped and closed-bite shaped dental CBCT datasets. According to these experiments, both the single-slice and thickened panoramic radiographs show the whole dentition without superimposing other dental structures. Moreover, these panoramic radiographs can be produced with thinner slices than those of other currently available methods; this technique leads to clearer images and easily identifiable anatomical structures. In addition, this method is automatic and only requires a few seconds. Although the resulting panoramic radiographs have slight geometric distortions and jagged artifacts, these flaws do not affect the overview of the whole dentition, which is the main application of the panoramic radiograph.

Future work will aim not only to reduce the geometric distortions generated by developing the panoramic curved surface but also to reduce normal estimation errors to improve the quality of thickened panoramic radiograph.
